# Research on skeletal muscle impact injury using a new rat model from a bioimpact machine

**DOI:** 10.3389/fbioe.2022.1055668

**Published:** 2022-11-14

**Authors:** Jun Liu, Zhikang Liao, Jingkun Wang, Hongyi Xiang, Xiyan Zhu, Xingping Che, Yuqian Tang, Jingru Xie, Chengyi Mao, Hui Zhao, Yan Xiong

**Affiliations:** ^1^ Department of Orthopedics, Daping Hospital, Army Medical University, Chongqing, China; ^2^ Institute for Traffic Medicine, Daping Hospital, Army Medical University, Chongqing, China; ^3^ Department of Pathology, Daping Hospital, Army Medical University, Chongqing, China

**Keywords:** impact injury, muscle contusion, animal model, repair, mechanisms, biomechanics

## Abstract

**Introduction**: Skeletal muscle impact injury occurs frequently during sports, falls, and road traffic accidents. From the reported studies on skeletal muscle injury, it is difficult to determine the injury parameters. Therefore, we developed a new model of gastrocnemius impact injury in rats with a bioimpact machine, with which the experimental operation could be conducted in feasibility from the recorded parameters. Through this novel model, we study the skeletal muscle impact injury mechanisms by combining temporal and spatial variation.

**Methods:** The gastrocnemius of anesthetized rats was injured by a small pneumatic-driven bioimpact machine; the moving speed and impact force were determined, and the whole impact process was captured by a high-speed camera. We observed the general condition of rats and measured the changes in injured calf circumference, evaluating calf injuries using MRI, gait analysis system, and pathology at different times after the injury.

**Results:** The gastrocnemius was injured at an impact speed of 6.63 m/s ± 0.25 m/s and a peak force of 1,556.80 N ± 110.79 N. The gait analysis system showed that the footprint area of the RH limb decreased significantly on the first day and then increased. The calf circumference of the injured limb increased rapidly on the first day post-injury and then decreased in the next few days. MRI showed edema of subcutaneous and gastrocnemius on the first day, and the area of edema decreased over the following days. HE staining showed edema of cells, extensive hyperemia of blood vessels, and infiltration of inflammatory cells on the first day. Cell edema was alleviated day by day, but inflammatory cell infiltration was the most on the third day. TEM showed that the sarcoplasmic reticulum was dilated on the first day, the mitochondrial vacuolation was obvious on the second day, and the glycogen deposition was prominent on the fifth day.

**Conclusion:** In our experiment, we developed a new and effective experimental animal model that was feasible to operate; the injured area of the gastrocnemius began to show “map-like” changes in the light microscope on the third day. Meanwhile, the gastrocnemius showed a trend of “edema-mitochondrial vacuolation-inflammatory cell aggregation” after impact injury.

## 1 Introduction

**FIGURE 1:**
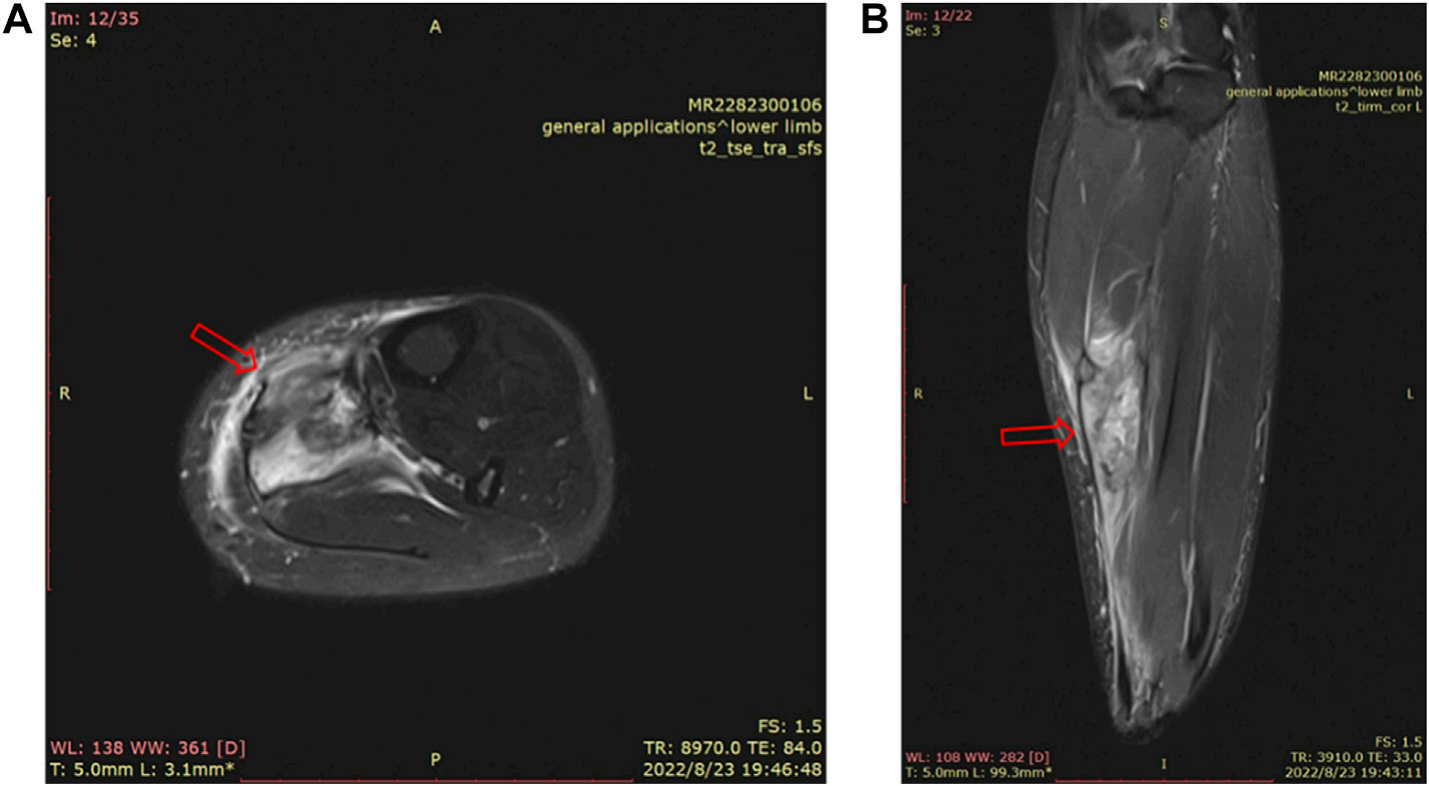
MRI of a 35-year-old female’s left calf. There is a mass-like high signal shadow in the flounder and posterior tibial muscles, a small amount of fluid in the intermuscular space, thickening and swelling of the surrounding fascia, and edema in the subcutaneous fat layer **(A)**. MRI image of the cross section of the left calf. **(B)**. MRI image of the sagittal plane of the left calf.

Skeletal muscle is one of the most vital tissues in humans. It comprises approximately 40%–50% of body mass, playing an essential role in physical activities (Frontera and Ochala, 2015; Zheng et al., 2019a; Yu et al., 2019; Cezar et al., 2016). Because the skeletal muscle’s anatomical location is relatively superficial, impacts and injuries often occur in daily life (Li et al., 2021). SMI (skeletal muscle injury) is observed frequently in clinics (Ekstrand et al., 2011; Flores et al., 2018; López-Valenciano et al., 2020; Portillo et al., 2020; Shou et al., 2021). Inflammation, pain, and dysfunction after SMI can lower the quality of life of patients. Mechanical injury accounted for more than half of all SMIs, which can be divided into three categories, strain, contusion, and tear (Souza and Gottfried, 2013), while contusion occupies the largest proportion of all types of SMIs in clinics (Flores et al., 2018; Dantas et al., 2017), more than 90% (Hartmann et al., 2020). Contusion is caused by rapid and strong compression force, usually from direct collision or falling, and often occurs in contact sports (such as basketball, football, and handball) (Cervaens et al., 2018; Vidoni et al., 2018; Hartmann et al., 2020; Ishøi et al., 2020) and traffic accidents (Yan, 2018; Lee et al., 2021). Figure 1 shows a 35-year-old female whose left calf suffered from a contusion. The pathogenesis of SMI has not been clearly elucidated due to different factors such as the environment, the magnitude/direction of external force, and the state of limbs when contusion occurs. In particular, when it is complicated with a fracture or neurovascular injury, SMI is often ignored or placed in a secondary position (Yan, 2018); incomplete repair after SMI, for chronic inflammation and scar, often leads to sequelae (limb pain, incomplete functional recovery, etc.), which has become the main problem and challenge in clinics (Chiu et al., 2020; Sakamoto, 2021; Yamamoto et al., 2021).

In the research of SMI, it is a major link in the relevant research process to accurately establish or duplicate animal injury models consistent with human diseases (Yan and Tang, 2021). The controllability, stability, and repeatability of animal models are directly related to the scientificity of experimental models and results. At present, SMI is mainly carried out by heavy falling (Bai et al., 2017; Wu et al., 2020; Ren et al., 2021; Yuan et al., 2021), toxin injection (Cezar et al., 2016; Guardiola et al., 2017; Cho et al., 2020; De Micheli et al., 2020), and ischemia–reperfusion injury (Raimondo and Mooney, 2018). Since heavy falling is the most widely applied model, it is more similar to the process of human injury, but the mass, height, geometry, and contact area of the heavy fall must be defined in the experiment (Souza and Gottfried, 2013).

In our study, therefore, we first developed a rat gastrocnemius injury model caused by a small pneumatic-driven bioimpact machine, which overcame many limitations of the heavy falling model. From the new animal model, we continuously observed the temporal and spatial changes of the gastrocnemius after impact injury and tried to explain the relationship between the local microscopic changes of the gastrocnemius and its macroscopic functional changes at every stage after the injury.

## 2 Materials and methods

### 2.1 Animal preparation

A total of 54 adult male Sprague–Dawley rats (obtained from the Experimental Animal Center of Daping Hospital, Chongqing, China), weighing 240 g ± 20 g, were randomly assigned into two groups, namely, the control group and the experimental group. Throughout the experiment, rats were kept in a temperature and humidity adjustable room under a 12-h light/12-h dark cycle with enough food and water. All animal procedures used in this study were approved by the Administration of Affairs Concerning Experimental Animals Guideline of the Army Medical University. The use of laboratory animals was in compliance with the guidelines of the National Institutes of Health. All animal experiments were approved by the Animal Use Subcommittee of the Army Medical University (Approval no. AMUWEC20223810).

### 2.2 Small pneumatic-driven bioimpact machine

The small pneumatic-driven bioimpact machine is composed of a driving unit, a control unit, and a 4-DOF adjustable frame. The driving unit is mainly composed of an AC (air compressor), a cylinder, an impactor, and a pipeline. AC compresses the external air and conveys it to the cylinder through the pipeline. The cylinder releases the compressed air and drives the impactor to move quickly, and then the latter impacts the target area. Impactors with different diameters can be selected according to a variety of experimental purposes or animal types. The control unit includes a solenoid valve and an air pressure regulating valve. The solenoid valve can control the start of the impact machine, while the air pressure regulating valve can adjust the output air pressure from the AC to the cylinder. A 4-DOF adjustable frame to support animals can move on horizontal and vertical planes in order to realize the accurate impact injury (Figure 1). In addition, the bioimpact machine also integrates a biomechanical recording system. The impact process and details was recorded by a high-speed camera (Phantom v12.1, Vision Research Inc., Wayne, United States), while the stress transmitted from the impactor to the animal limb was measured by a PVDF membrane pressure sensor (JYC10-3B, Jinzhou yikeda sensor Construction Co., Ltd., Liaoning, China). A DH5916 solid micro dynamic signal test and analysis system (DONGHUA TEST, China) is used to obtain signals.

### 2.3 Animal skeletal muscle contusion model

**FIGURE 2:**
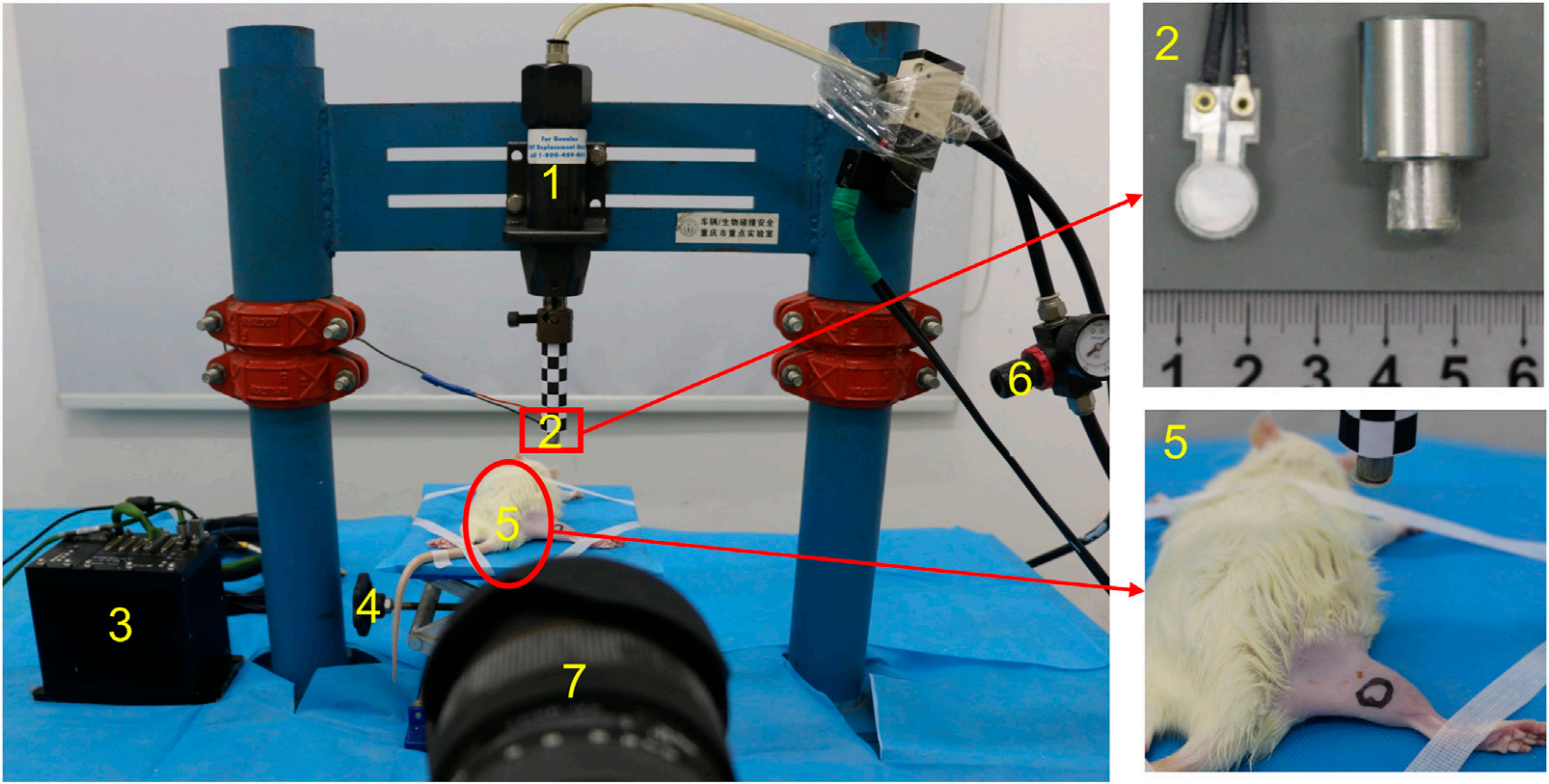
Photograph of the small pneumatic-driven bioimpact machine (1: cylinder, 2: impactor with a PVDF membrane pressure sensor, 3: signal test and analysis system, 4: 4-DOF adjustable frame, 5: rat, 6: air pressure regulating valve, and 7: high-speed camera).

We set the impactor with an appropriate diameter (d = 1 cm) and adjusted the output air pressure of the AC (*p* = 0.7 MPa). Rats were anesthetized by intravenous injection of pentobarbital (0.3%, 0.1 ml/100 g). Skin preparation with depilation cream on the RH (right hind) limb was performed. We selected the midsection of the gastrocnemius as the impact point, and circle marked with its midpoint as the center and diameter of 1 cm. Then, the anesthetized rats were put in the prone position on the 4-DOF adjustable frame (Figure 2). We adjusted the height of the frame and moved it on a horizontal plane so that the impactor is facing the gastrocnemius belly marking. The limbs and trunk of the rat were fixed with medical tape to prevent the animal from moving during the operation. We then adjusted the sampling rate of the high-speed camera to 1,000 frames/s and the sampling rate of the signal test and analysis system to 10 kHz.

### 2.4 Small animal MR

All MR imaging experiments were performed with a 7-T animal scanner (Biospin70/20 USR, Bruker BioSpin, Ettlingen, Germany). Rats were initially anesthetized by intravenous injection of pentobarbital (0.3%, 0.1 ml/100 g), and then anesthesia was maintained by 2% isoflurane in oxygen during MR scanning. The respiration and heart rate were monitored throughout the experiment. All animals were scanned with the T2 sequence (echo time: 45.00 ms, repetition time: 3411.685 ms, average: 6, field of view: 35 mm × 35 mm, matrix: 256 × 256, slice thickness: 8 mm, and scan time: 10 m 55 s 43 ms).

### 2.5 CatWalk gait analysis system

Before the injury, the animals were trained for one week with the CatWalk gait analysis system (CatWalk XT 10.0 noldus, Switzerland). The standard for training is that the animals pass through the channel continuously three times. The system was used to collect the gait parameters of rats passing through the channel 1 day before the injury and the first, second, third, and fifth days after the injury.

### 2.6 Histology and immunofluorescence

The samples were taken on the first, second, third, and fifth days of post-injury. Rats were killed immediately after intraperitoneal injection of excessive anesthetics, with six animals at each time point, four for optical microscopy and two for TEM (transmission electron microscopy). We cut the skin along the longitudinal axis of the RH limb, blunt-separated the superficial fascia such as skin and soft tissue, cut off the deep fascia, and exposed the gastrocnemius. The isolated gastrocnemius was washed with normal saline and fixed with 4% paraformaldehyde for 48 h and then dehydrated gradiently, embedded in paraffin, and sliced with a slicer (thickness of 5 mm). HE (hematoxylin–eosin) staining was performed following the routine procedure. To examine macrophage proliferation after the injury, the sections were incubated in 3% hydrogen peroxide to block endogenous peroxidase activity for 15 min. After three washes with PBS, the sections were blocked with PBS containing 10% BSA (bovine serum albumin) for 30 min at 37°C, followed by overnight incubation at 4°C in primary antibody. The primary antibodies used were rabbit antirat anti-CD68 antibodies (1:100 dilution, Abcam, United Kingdom). The next day, the slides were washed three times with PBS for 5 min each time and then incubated with the appropriate secondary antibody (donkey antirabbit IgG H&L antibody, Abcam, United Kingdom) for 4 h at room temperature. The slides were washed three times in PBS and counterstained with 4’,6-diamidino-2- phenylindole (DAPI; Solarbio, Beijing, China) for 2 min. Coverslips were applied with a mounting medium. Fluorescence was imaged on a BX43 upright fluorescence microscope system (Olympus, Tokyo, Japan); TEM: we take 0.1 cm × 0.1 cm × 0.2 cm tissue from the gastrocnemius, fix it with 2% glutaraldehyde for 24 h, rinse it with PBS three times, fix it with 1% osmium tetroxide for 1.5 h, rinse it twice, perform gradient dehydration with acetone (50%, 70%, 80%, 90%, and 100%), embed it with epoxy resin, carry out ultrathin slicing with an ultrathin slicer, and stain it with 2% uranyl acetate and 2% lead citrate. The ultrastructural changes in skeletal muscle in each group were observed by TEM.

### 2.7 Statistical analysis

All data are presented as mean ± standard deviation (‾X ± SD), unless otherwise indicated. The footprint area and calf circumference of the affected side in different periods were compared by ANOVA of repeated measurement design. If the Mauchly sphericity test is obeyed, the subject internal effect test is adopted; if not, the multivariate test is adopted. Differences were considered significantly when *p* < 0.05.

## 3 Results

### 3.1 Experimental mechanical parameters

**FIGURE 3:**
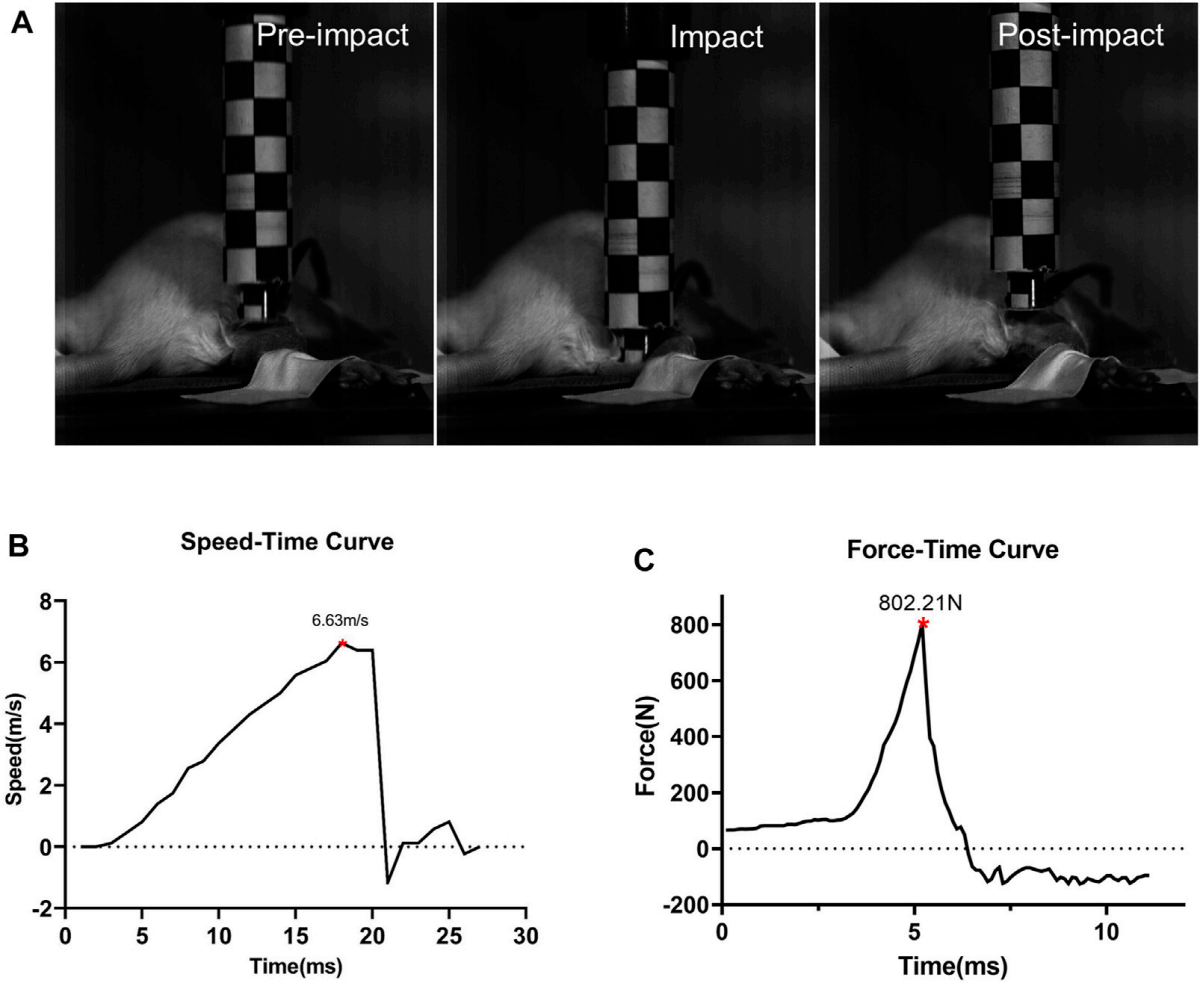
Impact process and impact-related parameters (*n* = 5). **(A)** Impact process. Details before, during, and after the impact. **(B)** Speed-time curve of the impactor. **(C)** Force curves detected by the sensor during impact.

After the solenoid valve is activated, the impactor moves downward rapidly, as shown by the high-speed camera (Figure 3A). When the maximum speed reaches about 6.63 m/s ± 0.25 m/s, it impacts the hind limb. During the impact, the tissue is compressed, lasting less than 10 ms, and the speed of the impactor drops rapidly to 0 m/s and moves upward. The duration of the whole impact process is extremely short, and the extrusion time of the animal’s hind limbs can be ignored (Figure 3B). The curve recorded from the PVDF membrane pressure sensor showed when the impactor contacts the lower limb of the animal, the force at the contact area rises rapidly, and the peak value is about 1,556.80 N ± 110.79 N. After climbing to the peak, it decreases rapidly.

### 3.2 General conditions of animals

**FIGURE 4:**
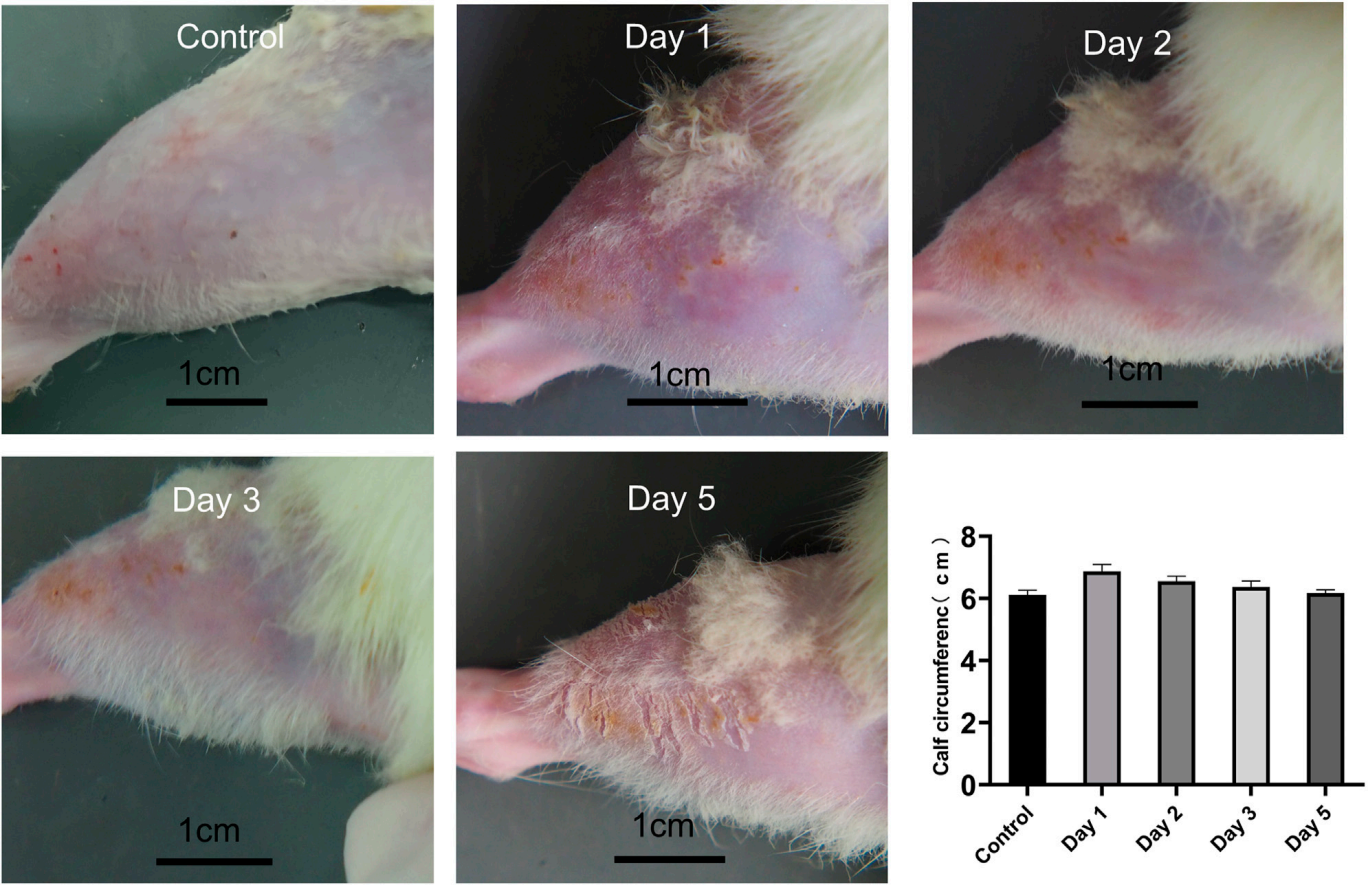
Calf condition of rats at different time points, and calf circumference at different time points (*n* = 10). Scale bar = 1 cm.

All rats were examined immediately after the impact injury. All survived, and no fracture was found in the RH limbs. Congestion and edema began to appear locally after the impact. On the day of injury, the rats were in a poor mental state after waking up from anesthesia, curled up in the corner of the cage, and their activities were reduced. The food intake of some experimental rats was reduced compared with that before the injury, and there was no significant change in urination and defecation. On the first, second, and third days of post-injury, the mental state of the experimental rats improved, and their daily activities increased than the previous day. On the fifth day after the injury, the activity of rats almost recovered. The edema of rats’ lower limbs was the most serious on the first day (6.87 cm ± 0.23 cm) and gradually decreased on the second (6.55 cm ± 0.16 cm), third (6.36 cm ± 0.20 cm), and fifth day (6.17 cm ± 0.11 cm) (*p* < 0.05, *n* = 10) (Figure 4).

### 3.3 Gait analysis

**FIGURE 5:**
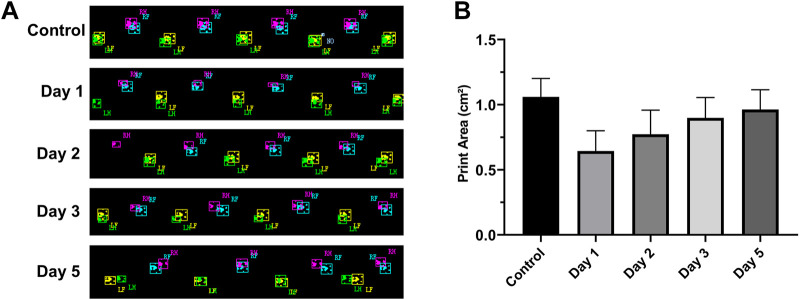
RH footprints at different times and their change trend (*n* = 10, RF = right front, LF = left front, RH = right hind, and LH = left hind). **(A)** Footprints at different time points. **(B)** Footprint area–time line chart. The footprint area of rats decreased significantly on the first day after the injury and then increased slowly on the next few days.

After the rats were injured, we selected the RH footprint area for analysis (Figure 5A). The footprint area on the first day (0.64 cm^2^ ± 0.164 cm^2^), the second day (0.77 cm^2^ ± 0.18 cm^2^), the third day (0.90 cm^2^ ± 0.164 cm^2^), and the fifth day (0.96 cm^2^ ± 0.154 cm^2^) after the injury decreased significantly compared with that before injury (1.06 cm^2^ ± 0.14 cm^2^) (*p* < 0.05, *n* = 10) (Figure 5B). The footprint area of rats decreased most significantly on the first day after the injury. With the extension of time, the footprint area increased slowly and did not return to the preinjury level till the fifth day.

### 3.4 7-T MRI

**FIGURE 6:**
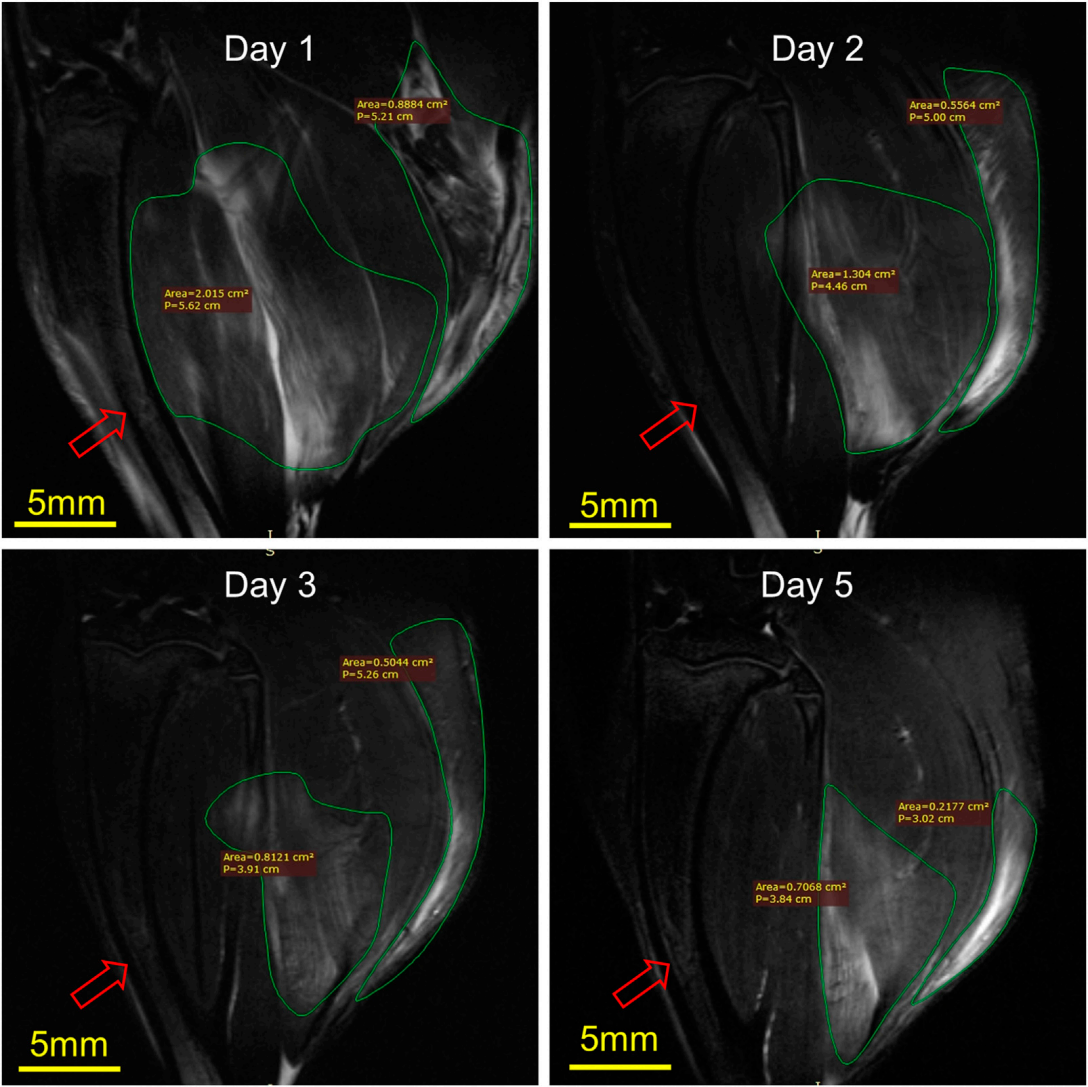
Representative MRI of rat RH limbs at different times after the injury (*n* = 4). Scale bar = 5 mm. The same tibial (red arrow) plane of the same rat was selected for observation. On the first day, the areas of muscle edema and subcutaneous edema were 2.015 cm^2^ and 0.89 cm^2^, respectively, the second day were 1.30 cm^2^ and 0.56 cm^2^, the third day were 0.81 cm^2^ and 0.50 cm^2^, while on the fifth day, they were 0.70 cm^2^ and 0.22 cm^2^.

Figure 6 illustrates the MRI of rats’ RH limbs at different times after the injury. Obvious skeletal muscle edema and subcutaneous edema can be seen on the first day after the injury. Irregular clumped hypointensity was observed within the gastrocnemius, and the fasciculation pattern disappeared, suggesting bleeding. The scope of edema and subcutaneous edema on the second day after the injury is lower than that on the first day. The subcutaneous edema continued to decrease on the third day. On the fifth day, we could observe that the range of abnormal signals was reduced to half of that on the first day and the muscle bundle morphology was largely restored. In general, the area of skeletal muscle edema and subcutaneous edema decreased gradually over time.

### 3.5 Histology and immunofluorescence analysis

**FIGURE 7:**
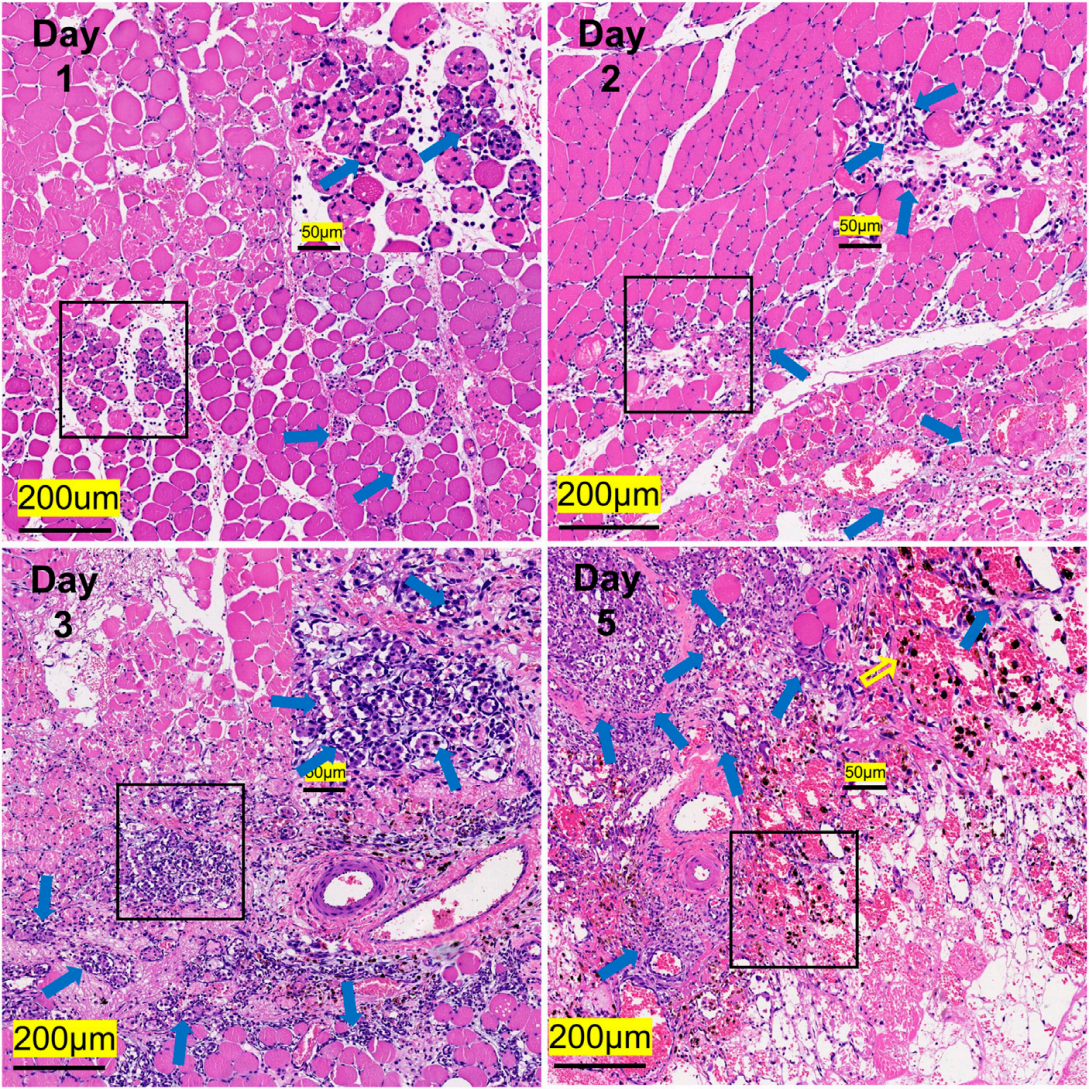
Representative HE staining images of the gastrocnemius at different times after the injury (*n* = 20). Scale bar = 200 μm. (Blue hollow arrows indicate inflammatory cells, and the yellow indicates hemosiderin).

**FIGURE 8:**
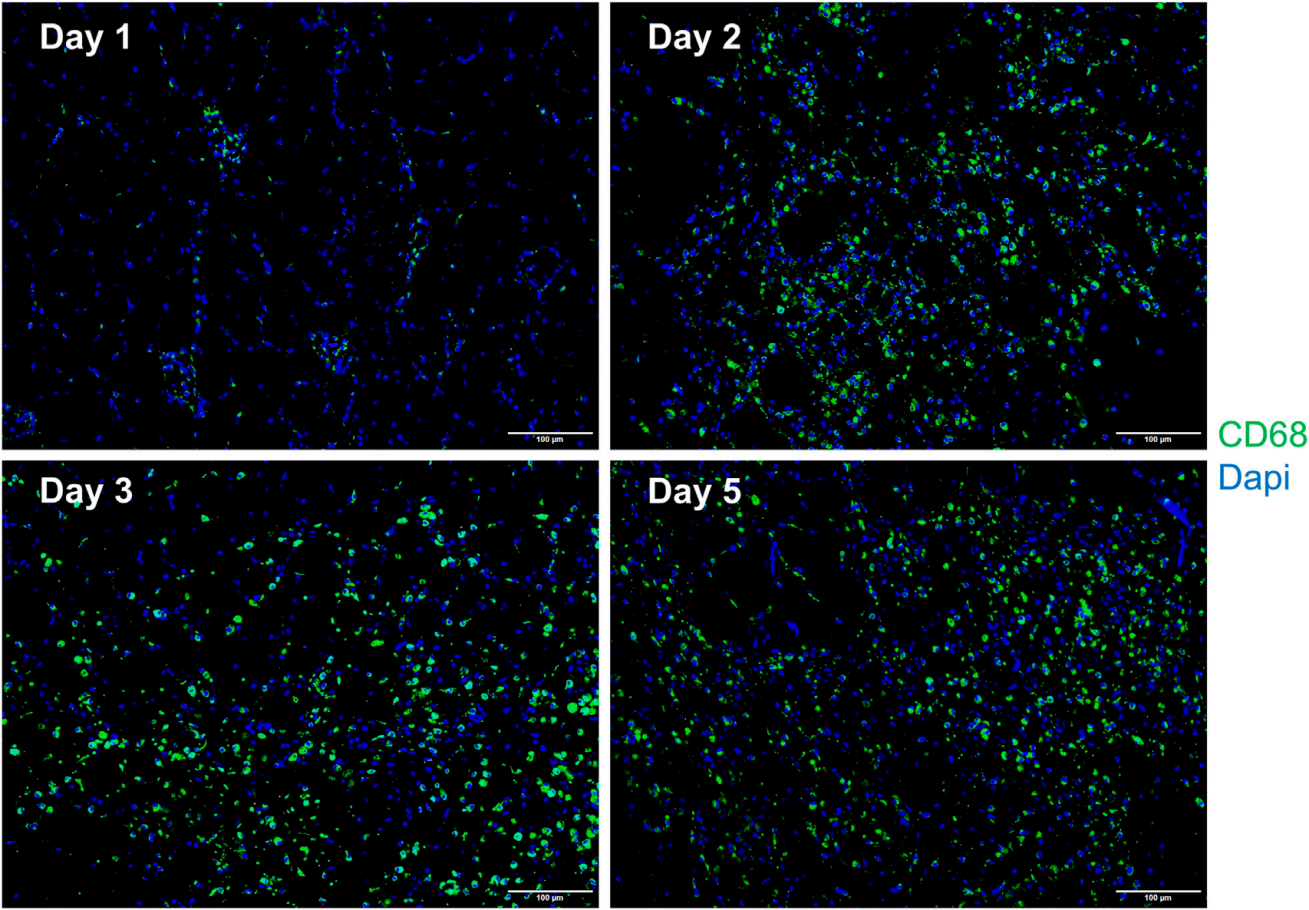
Representative merged macrophage IF images of the gastrocnemius at different times after the injury (*n* = 10). Blue dots represent the nuclei, and green dots represent the macrophages. Scale bar = 100 μm. A small number of macrophages can be seen on the first day. The macrophages increase on the second day, reach the peak on the third day, and the fifth day is similar to the third day.

In comparison with the control, it was found that a significant alternation in HE staining of the gastrocnemius occurred for the experimental rats (Figure 7). Interstitial edema with fibrinous exudation occurred on the first day, with local striated muscle cell rupture with degeneration. A few to moderate inflammatory cells appear around the injury. Macrophages phagocytose damaged muscle cells. At the injured site, most of the skeletal muscle cytoplasm is homogenized and lightly stained. On the third day, the interstitial edema and inflammatory cell infiltration were further aggravated, and the inflammatory cells reached their peak. On the fifth day, a large area of interstitial edema was seen, accompanied by moderate fibrinous exudation. A large number of inflammatory cells infiltrated near the injury target. Hemosiderin calms around the injury. Plenty of macrophages appear in muscle cells and engulf injured muscle cells (Figure 7). The macrophages labeled with CD68 began to proliferate after the injury and reached their peak on the third day. The number of macrophages on the fifth day is similar to that on the third day (Figure 8). Table 1 shows the grading of pathological changes.

**TABLE 1:** Pathological comparison of muscle injury at different times. [Relevant evaluation criteria were based on the method described previously (Wu et al., 2020)].

	Day 1	Day 2	Day 3	Day 5
Degree of interstitial edema	+	+	++	+++
Degree of cellulose exudation	+	+	+	++
Range of muscle tissue degeneration	+	+	+	+
Degree of inflammatory cell infiltration	+	+	+++	+++
Degree of macrophage infiltration	+	+	+++	+++
Muscle tissue phagocytosis	+	+	+	+

### 3.6 TEM observation

**FIGURE 9:**
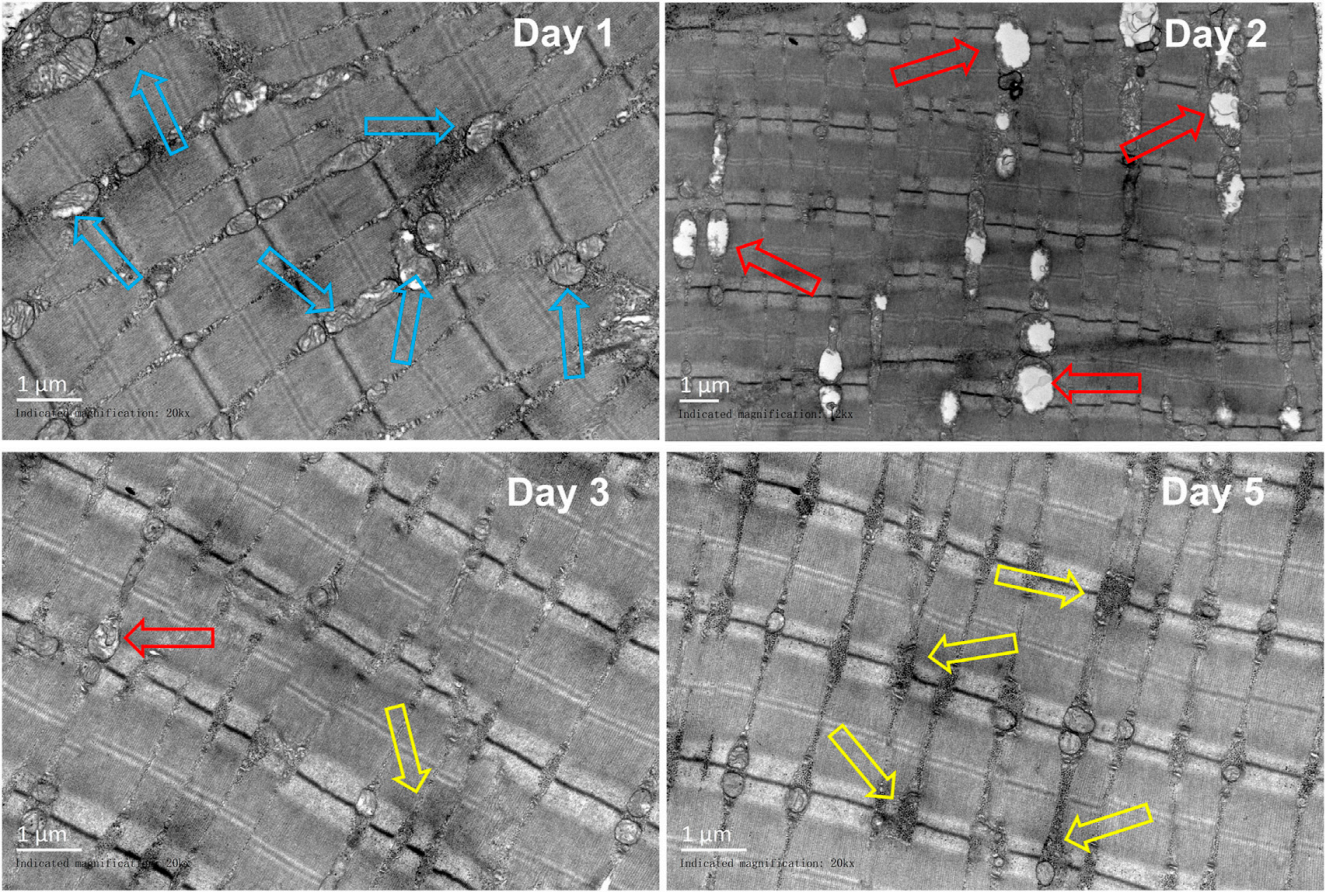
Representative TEM images of the gastrocnemius at different periods (*n* = 10). Scale bar = 100 μm. Blue hollow arrows indicate the sarcoplasmic reticulum, red arrows indicate the mitochondrion, and yellow arrow indicates the glycogen).

In comparison to those in the control group, obvious sarcoplasmic reticulum expansion was observed on the first day after the injury. The vacuolation of mitochondria was obvious on the second day. On the third day, a small amount of sarcoplasmic reticulum expansion can be seen, and glycogen deposition begins to appear near the sarcoplasmic reticulum. On the fifth day, a great amount of glycogen was deposited near the sarcoplasmic reticulum (Figure 9).

## 4 Discussion

Experimental methods varied from the published studies regarding skeletal muscle impact injury. Heavy falling is widely used in the study of impact injury, regardless of whether there is skeletal muscle exposure or not. Most studies use single impact (Lee et al., 2019; Hsu et al., 2020). Early heavy-falling techniques used a smooth impact plane and modulated the height of the fall to injure specific muscles (Crisco et al., 1996; Delos et al., 2014; Zhou et al., 2018). As a conventional SMI model, the heavy falling technique has the advantages of being noninvasive, adjustable, and easy to operate (Deane et al., 2014; Zheng et al., 2019b). However, the limitations of this damage model are presented conspicuously. On one hand, the studies may result in the poor fixation of the target skeletal muscle site, an uneven degree of injury, and skin damage or fracture. On the other hand, according to the law of conservation of energy, the parameters of the injury potential (mass and height) are different in several contusion models, theoretically leading to differences in impact response and tissue damage. To date, there has been a lack of studies of impact responses in the damaged gastrocnemius separated from the underlying bone using heavy falling techniques with variable parameters (Yan and Tang, 2021).

In the present study, we pioneered the use of a pneumatic-driven impactor to model blunt gastrocnemius injuries. This model overcomes the shortcomings of the previously reported heavy falling. In our experiment, we used the biomechanical recording system to record the details of the impact, calculating the mean and standard deviation of the impact force and impact velocity and plotting the corridor of the impact parameters. We found that the parameter distribution regime was relatively narrow, suggesting that the injury parameters had good consistency. The consistency of experimental parameters ensures the consistency of injuries. Furthermore, skeletal muscle is an elastic tissue. In traditional animal models, when heavy objects fall on skeletal muscle, inevitably, the heavy objects will rebound and fall, causing skeletal muscle “secondary fall” damage. Our bioimpact machine will stay away from skeletal muscle after contact with it to avoid “secondary injury” perfectly. Moreover, in our model, the velocity of the impactor can reach 6.63 m/s ± 0.25 m/s, while in the traditional models, the impact velocity of the heavy object varied from 1.78 m/s to 5.19 m/s (Minamoto et al., 1999; Khattak et al., 2010; Souza and Gottfried, 2013; Dantas et al., 2017; Russ et al., 2018; Yan et al., 2019). Furthermore, no reports in the literature have reported the tissue crash time of traditional models to date, but in our study, the tissue crashing time is less than 10 ms, which is certainly much less than that in traditional animal models.

Ultrasound is traditionally considered the preferred method to diagnose SMI in the clinic because it is feasible to operate. While there is an obvious disadvantage, it is highly dependent on the experience of sonographers (De Smet and Best, 2000; Slavotinek et al., 2002; Järvinen et al., 2007). Recently, MRI has replaced ultrasound in the imaging of many musculoskeletal diseases. It can accurately confirm/rule out the presence of muscle damage and also provides a detailed characterization of the lesion and is sometimes considered to be somewhat oversensitive. (Järvinen et al., 2007). It is the first time that 7-T MRI has been used to detect SMI in animals, from which the injury alternation may be observed clearly, with which the muscle pattern may be determined quantitatively.

At the microscopic level, HE staining is a commonly used method for histological detection. Wu et al. (2020) established a rabbit model of acute SMI and found that the muscle fibers were significantly swollen and partially ruptured on the first day after modeling. The inflammatory response further escalated on the third day, and a large number of inflammatory cell infiltration, fibrinoid, and vacuolar-like changes were seen under the microscope. Inflammatory cells began to decrease on the fifth day, while cell edema and inflammatory reactions began to reduce, which is similar to our results. Furthermore, in our experiment, the inflammation peaked on the third day and then gradually subsided. In addition, our study further found that the injured area of the gastrocnemius began to show “map-like” changes on the third day (Figure 7). Damaged skeletal muscle cells (cells in the map) are less stained than normal skeletal muscle cells (cells outside the map). Macrophages gather around the injury from other sites, phagocytizing damaged and necrotic skeletal muscle cells, thus forming the boundary of the map. The phagocytic skeletal muscle cells showed “vacuolar” changes. Until the fifth day, the “map-like” changes were still clearly visible, and unlike on the third day, hemosiderin began to appear in the local part of the injury.

Yuan et al. (2021) observed the changes in the rabbit gastrocnemius after contusion by TEM. It was found that on the first day of post-injury, the myofibril was disordered, dissolved, and disappeared, and the mitochondrial crest dissolved and disappeared. On the second day, the arrangement of muscle filaments was twisted, and the rest were similar to those on the first day. The arrangement of myofibril was disordered and loose, the myofilament was twisted, and the mitochondria were vacuolated on the third day, while on the fifth day, the distortion of myofibril was alleviated, the arrangement of myofibril was disordered, the mitochondria enlarged, the structure partially recovered, the mitochondrial crest existed, and the vacuoles decreased. Luo et al. (2018) found that on the third day after acute blunt trauma of the gastrocnemius in rats, the mitochondria were obviously swollen, and the structure was abnormal, with cristae degeneration, vacuolization, and mild expansion of the sarcoplasmic reticulum. On the fifth day, a large area of the mitochondria was swollen. In our model, sarcoplasmic reticulum dilation was seen on the first day after the injury, mitochondrial cristae disappeared, obvious vacuolation was seen on the second day, and glycogen deposition was seen on the fifth day.

The repair of SMI can be divided into three stages: the inflammation stage, the satellite cell activation/differentiation stage, and the maturation stage, in which the newly formed muscle fibers are remolded (Tidball, 2011; Turner and Badylak, 2012; Le Moal et al., 2017; Liu et al., 2018). These three processes of SMI are connected closely and have overlapping phases; therefore, it is difficult to clearly distinguish them. The inflammatory reaction after SMI is triggered by necrotic muscle fibers. Neutrophils are the first inflammatory cells to be collected from the injured muscle. Neutrophils can be detected at the injured site 1 h after the injury, reaching a peak 6 h–24 h after the injury and then declining rapidly (Turner and Badylak, 2012; Liu et al., 2014). The M1 macrophages invaded the injured site about 24 h after the injury, peaked at 2–3 days after the injury, and then gradually decreased. Then, macrophages experienced phenotypic and functional changes and turned into anti-inflammatory M2 macrophages characterized by the production of IL-4 and IL-10. The M2 macrophages peaked about 4 days after the injury and continued to the remodeling stage of skeletal muscle repair (Xiao et al., 2014; Baghdadi and Tajbakhsh, 2018). Macrophages are the major inflammatory cells after impact injury. Their phenotypes change at different times. They can not only regulate inflammation but also participate in muscle regeneration, extracellular matrix remodeling, and angiogenesis (Wang and Zhou, 2022).

After gastrocnemius impact injury, the impact of local skeletal muscle ultrastructure and peripheral vascular damage causes edema and pain in the lower limbs. Cellular edema leads to local oxygen supply deficiency and cellular hypoxia, which causes sarcoplasmic reticulum expansion, mitochondrial swelling, and vacuolization (Hartmann et al., 2020). In addition, the sarcoplasmic reticulum is an important Ca^2+^ reservoir for skeletal muscle cells, regulating muscle contraction by storing and releasing Ca^2+^, which plays a key role in mitochondrial swelling (Javadov et al., 2018). Therefore, we consider that the changes associated with the sarcoplasmic reticulum and mitochondria after impact injury may be related to the dysregulation of Ca^2+^ metabolism.

The function always adapts to structure, and the same is true in our experiments. Pain in the limb leads to a reduction in the contact area of the affected foot with the CatWalk plate, a shortening of the contact time, and a prolongation of the limb off the plate, which is clinically referred to as “claudication” and is thus reflected in the CatWalk parameters as a reduction in the footprint area (Wu et al., 2012). In addition to pain, dysregulation of Ca^2+^ can also cause skeletal muscle contraction and diastolic disorders, perhaps an important link in causing gait changes in rats.

Our experiments showed that the edema of lower limbs was serious on the first day after the injury, the vacuolation of mitochondria reached its peak on the second day, and the inflammatory cell infiltration was the most on the third day, showing an overall trend of “edema-mitochondrial vacuolation-inflammatory cell aggregation.” In terms of causing gait changes, dysregulation of Ca^2+^ metabolism may be another important factor besides pain. In short, our findings may help advance the study of mechanisms of blunt injury and the repair of skeletal muscles.

## Data Availability

The original contributions presented in the study are included in the article/Supplementary Material; further inquiries can be directed to the corresponding authors.
