# Supplementary material for: Research on skeletal muscle impact injury using a new rat model from a bioimpact machine
Source: Front Bioeng Biotechnol. 2022 Nov 14;10:1055668. doi: 10.3389/fbioe.2022.1055668 (PMC9701740; doi:10.3389/fbioe.2022.1055668)
Supplement: Supplementary file 1 [file Table1.docx]

Supplementary table

|  | Control  ‾X±SD | Day 1  ‾X±SD | Day 2  ‾X±SD | Day 3  ‾X±SD | Day 5  ‾X±SD | F | p |
| --- | --- | --- | --- | --- | --- | --- | --- |
| Calf circumference（cm） | 6.11±0.15 | 6.87±0.23 | 6.55±0.16 | 6.36±0.20 | 6.17±0.11 | 126.53 | 0.000 |

Table1. Statistical results of changes in calf circumference at different times.

Table2. Statistical results of RH footprints at different times

|  | Control  ‾X±SD | Day 1  ‾X±SD | Day 2  ‾X±SD | Day 3  ‾X±SD | Day 5  ‾X±SD | F | p |
| --- | --- | --- | --- | --- | --- | --- | --- |
| RH footprints (cm^2^) | 1.06±0.14 | 0.64±0.16 | 0.77±0.18 | 0.90±0.16 | 0.96±0.15 | 39.30 | 0.000 |
