# Supplementary figures and images for: Research on skeletal muscle impact injury using a new rat model from a bioimpact machine
Source: Front Bioeng Biotechnol. 2022 Nov 14;10:1055668. doi: 10.3389/fbioe.2022.1055668 (PMC9701740; doi:10.3389/fbioe.2022.1055668)

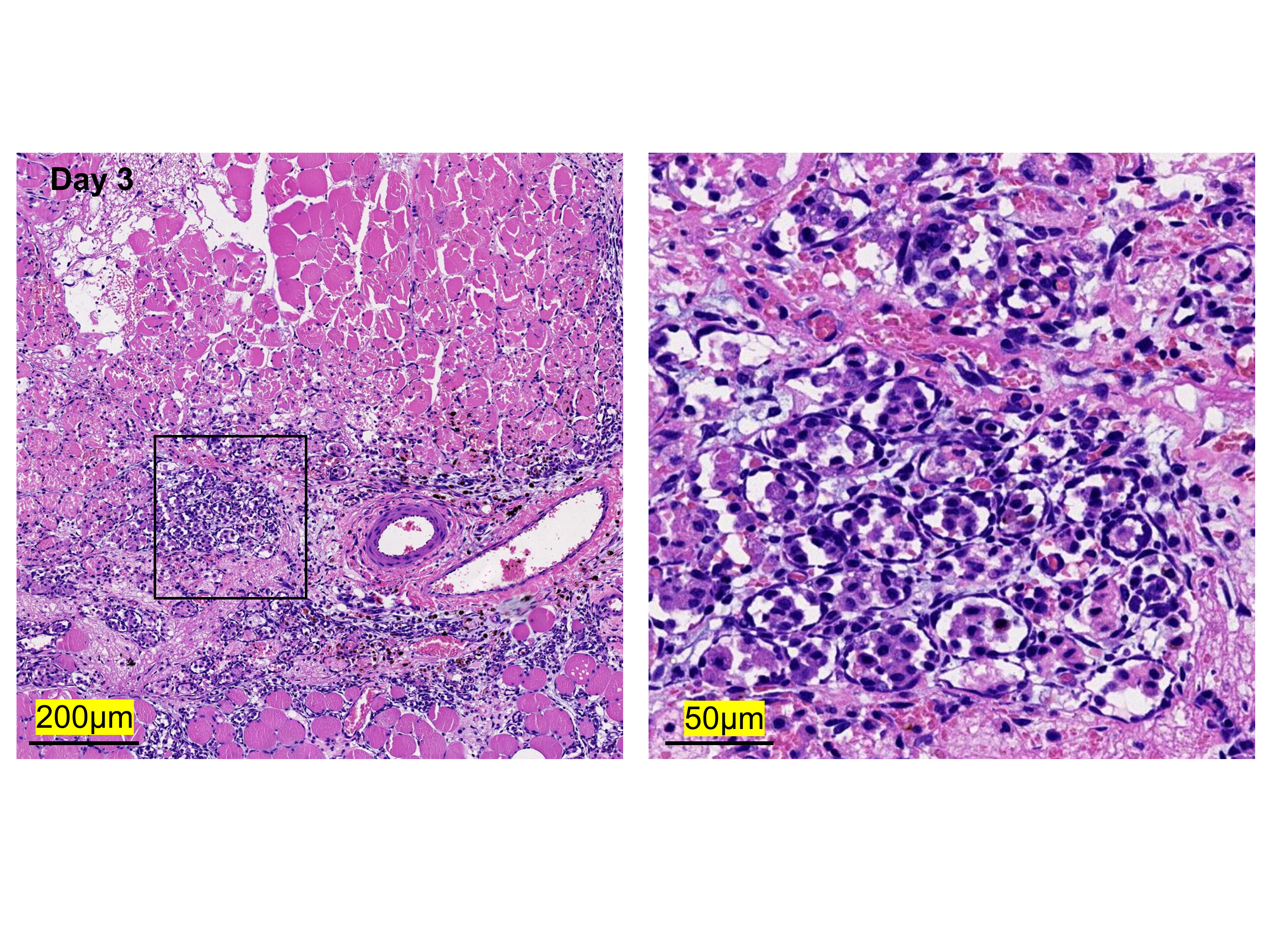

Supplement: Supplementary file 2 [file Image3.TIF]

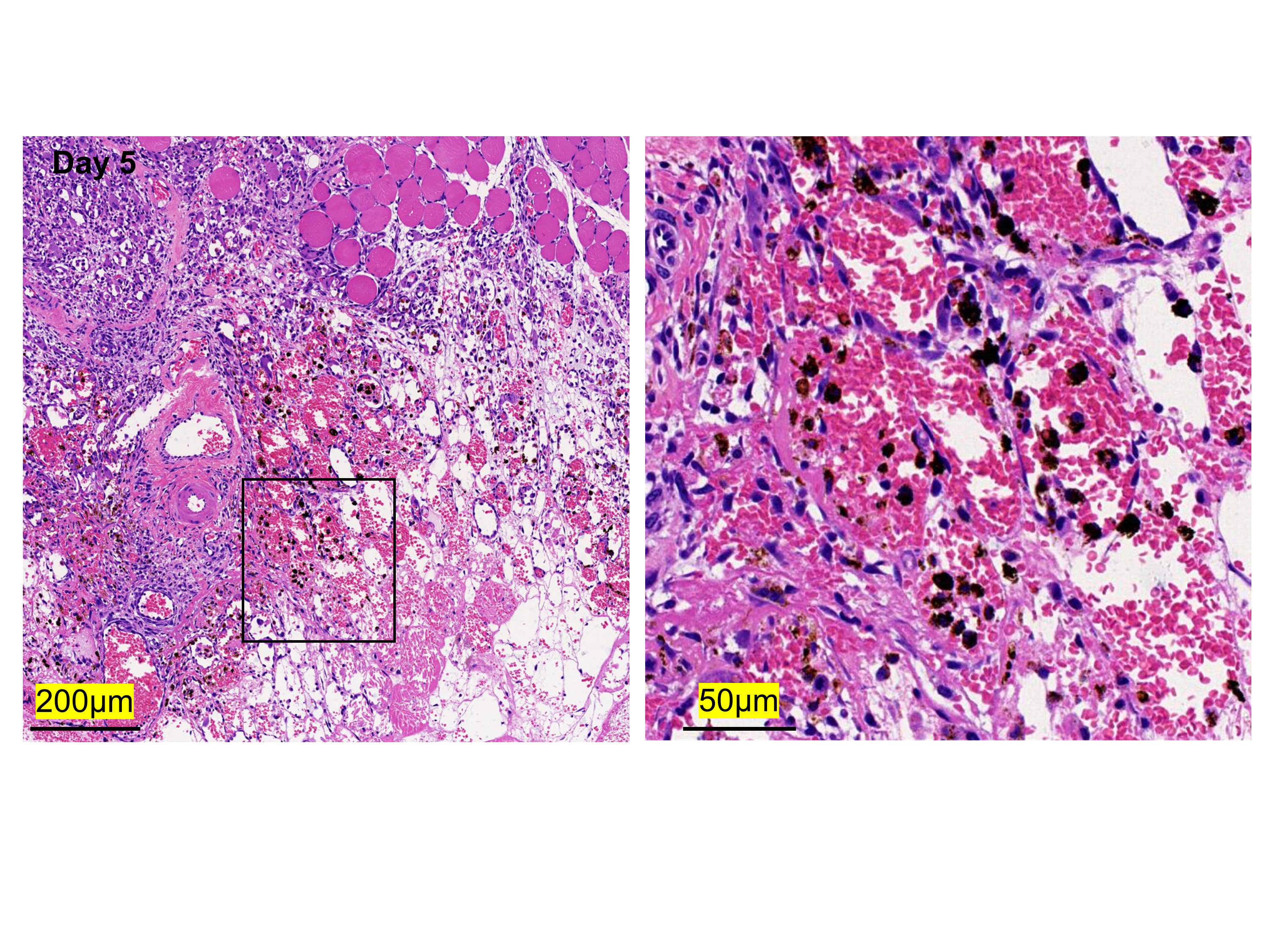

Supplement: Supplementary file 3 [file Image4.TIF]

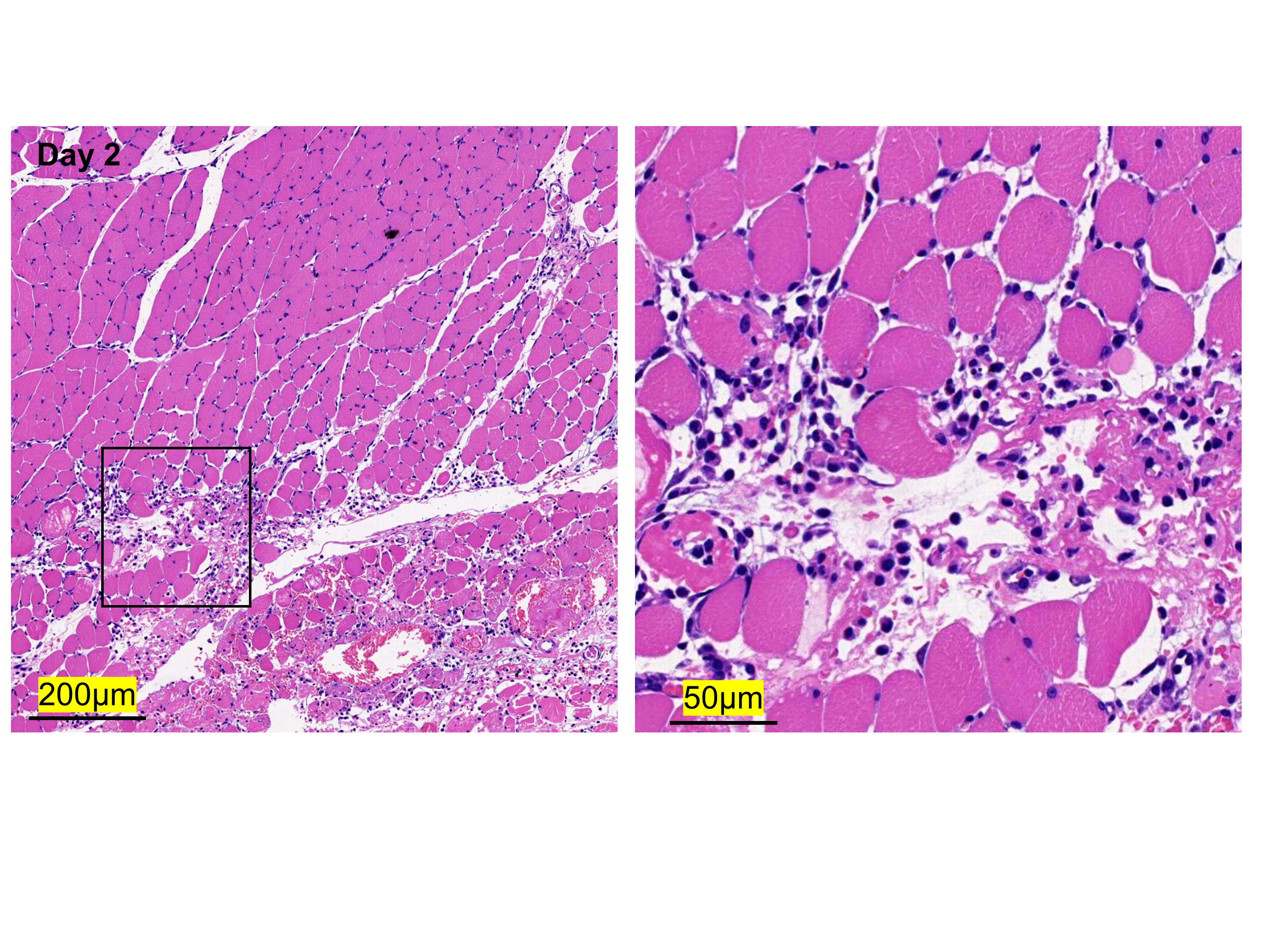

Supplement: Supplementary file 4 [file Image2.TIF]

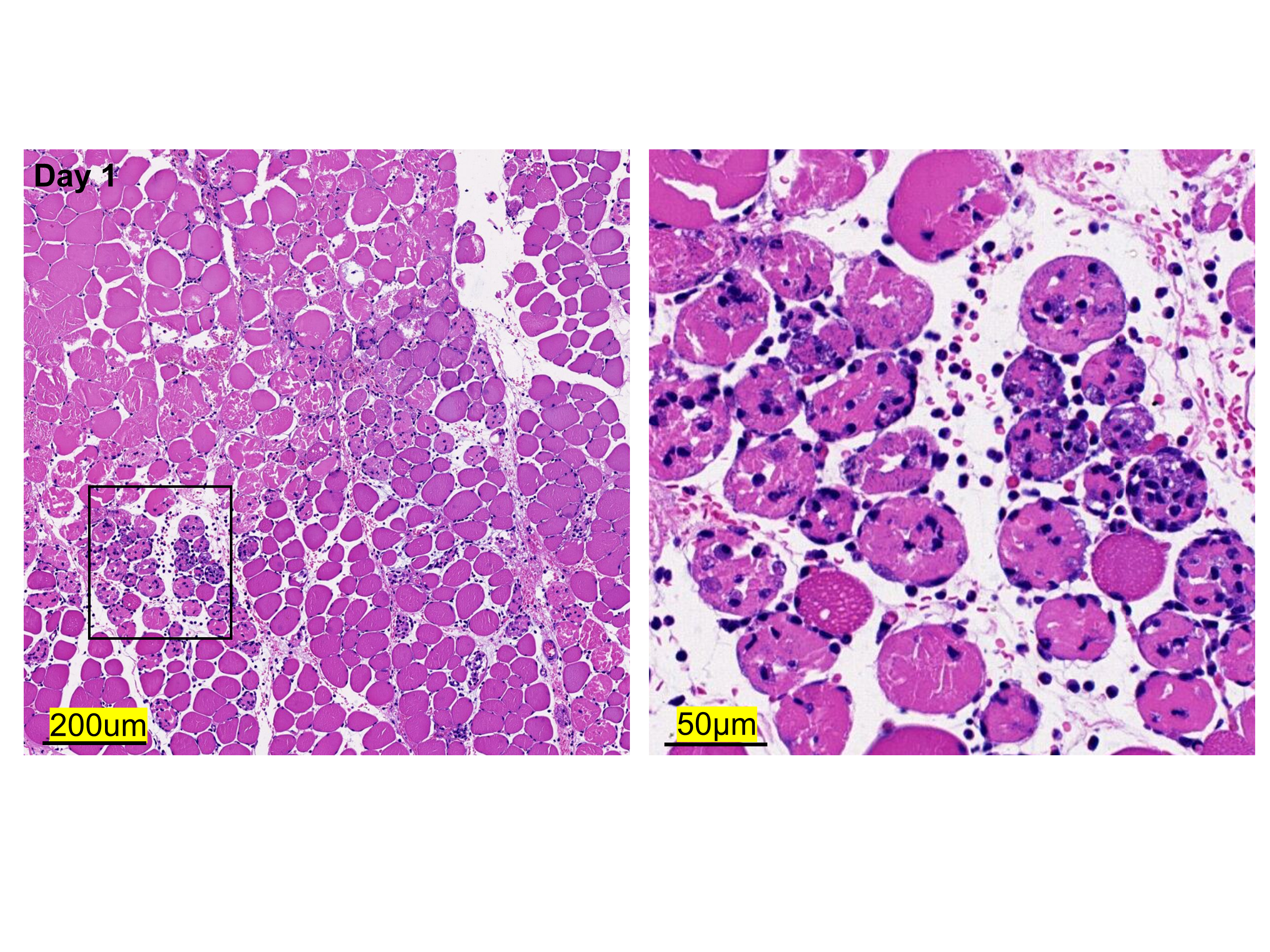

Supplement: Supplementary file 5 [file Image1.TIF]
